# Analysis and validation of m6A regulatory network: a novel circBACH2/has-miR-944/HNRNPC axis in breast cancer progression

**DOI:** 10.1186/s12967-021-03196-4

**Published:** 2021-12-24

**Authors:** Wenchang Lv, Yufang Tan, Mingchen Xiong, Chongru Zhao, Yichen Wang, Min Wu, Yiping Wu, Qi Zhang

**Affiliations:** Department of Plastic and Cosmetic Surgery, Tongji Hospital, Tongji Medical College, Huazhong University of Science and Technology, 1095 Jiefang Avenue, Wuhan, 430030 Hubei China

**Keywords:** Breast cancer, circular RNA, microRNA, m6A RNA methylation modulator

## Abstract

**Background:**

N6-methyladenosine (m6A), the most abundant and reversible modification of mRNAs in eukaryotes, plays pivotal role in breast cancer (BC) tumorigenesis and progression. Circular RNAs (circRNAs) can act as tumor promoters or suppressors by microRNA (miRNA) sponges in BC. However, the underlying mechanism of circRNAs in BC progression via regulating m6A modulators remains unclear.

**Methods:**

Prognostic m6A RNA methylation regulators were identified in 1065 BC patients from The Cancer Genome Atlas (TCGA) project. Differentially expressed (DE) miRNAs and DE circRNAs were identified between BC and normal samples in TCGA and GSE101123, respectively. MiRNA-mRNA interactive pairs and circRNA-miRNA interactive pairs were verified by MiRDIP and Circular RNA Interactome. GSEA, KEGG, and ssGSEA were executed to explore the potential biological and immune functions between HNRNPC-high and HNRNPC-low expression groups. qRT-PCR and Western blot were used to quantify the expression of HNRNPC and circBACH2 in MCF-7 and MDA-MB-231 cells. The proliferation of BC cells was assessed by CCK-8 and EdU assay.

**Results:**

2 m6A RNA methylation regulators with prognostic value, including HNRNPC and YTHDF3, were identified in BC patients. Then, the regulatory network of circRNA-miRNA-m6A modulators was constructed, which consisted of 2 DE m6A modulators (HNRNPC and YTHDF3), 12 DE miRNAs, and 11 DE circRNAs. Notably, BC patients with high expression of HNRNPC and low expression of hsa-miR-944 were correlated with late clinical stages and shorter survival times. Besides, the results from the KEGG inferred that the DE HNRNPC was associated with the MAPK signaling pathway in BC. Moreover, the circBACH2 (hsa_circ_0001625) was confirmed to act as hsa-miR-944 sponge to stimulate HNRNPC expression to promote BC cell proliferation via MAPK signaling pathway, thus constructing a circBACH2/hsa-miR-944/HNRNPC axis in BC.

**Conclusions:**

Our findings decipher a novel circRNA-based m6A regulatory mechanism involved in BC progression, thus providing attractive diagnostic and therapeutic strategies for combating BC.

**Supplementary Information:**

The online version contains supplementary material available at 10.1186/s12967-021-03196-4.

## Background

Breast cancer (BC) is the most common female malignancy and is a major cause of cancer-related mortality in the US [1]. Despite improvements in diagnostics and therapeutic strategies for BC in recent decades, the prognosis and long-term survival of BC patients remains poor, which attribute to the molecular heterogeneity, high metastatic characteristics, and low detection rate of BC [2]. Therefore, it is important to explore new molecular biomarkers with high specificity and sensitivity of BC detection and treatment.

N6-methyladenosine (m6A) modification, the methylation of the adenosine base at the nitrogen-6 position of mRNA, is the most prevalent, abundant, and conserved internal co-transcriptional modification in eukaryotes. The impacts of m6A on RNA are determined by the dynamic interactions between m6A RNA methylation modulators, including m6A methyltransferases (writers), binding proteins (readers), and demethylases (erasers) [3]. Generally speaking, m6A is installed by methyltransferase complex that functions as m6A writers, consisting of METTL3, METTL14, RBM15, and ZC3H13 [4]. The m6A erasers, such as FTO, ALKBH3, and ALKBH5, are able to remove m6A modification to balance methylation and demethylation [5]. Furthermore, the reversible m6A modification also depends on the recognition by m6A-binding proteins, namely the YTH domain-containing family of proteins, which is denoted as readers [6, 7]. Emerging evidence indicates that m6A modifications are closely associated with tumorigenesis, tumor proliferation, differentiation, metastasis, and poor prognosis [8]. The writers, erasers, and readers of m6A RNA modification engage in the tumorigenesis and progression of BC. These BC-specific m6A modulators are potentially useful for serving as prognosis and therapy targets.

Circular RNAs (CircRNAs) are single-stranded RNA molecules, lacking a 5-prime cap and 3-prime poly-A tail and joining head to tail to create a covalently closed loop structure via back-splicing [9]. In BC, the most studied function of circRNA is acting as tumor promoters or suppressors by miRNA sponges. Besides, emerging studies have identified the roles of circRNAs serving as miRNA sponges in the regulation of m6A RNA methylation modulators in various cancers, including hepatocellular carcinoma [10], gastric cancer [11], and adrenocortical carcinoma [12]. However, few studies have comprehensively investigated roles in the interaction of circRNAs and m6A in BC. The identification of circRNA regulatory network related to m6A RNA methylation modulators will undoubtedly provide key clues for mechanism discovery and therapeutic targets for BC.

In the present study, we identified differentially expressed (DE) prognostic m6A RNA methylation modulators, circRNAs, and miRNAs between BC samples and normal tissues from The Cancer Genome Atlas (TCGA) projects and Gene Expression Omnibus (GEO) database, and then constructed the circRNA-miRNA-mRNA regulatory network. Based on the clinical characteristics and co-expression patterns of miRNA-m6A RNA modulators, it was speculated that BC patients with high expression of hsa-miR-944 and low expression of HNRNPC were significantly concerned with longer survival times than control. Importantly, circBACH2, also known as hsa_circ_0001625, was further confirmed to promote BC cell proliferation via acting as hsa-miR-944 sponge to regulate HNRNPC expression. Moreover, the circBACH2/hsa-miR-944/HNRNPC axis accelerated BC progression via the MAPK signaling pathway-dependent manner. Undoubtedly, these findings ﻿shed new light on how circRNAs regulate m6A RNA methylation modulators by directly binding to miRNAs, thereby ﻿providing new perspectives for the development of clinical diagnostic and therapeutic strategies against BC.

## Methods

### Data collection and processing of BC datasets

The RNA-seq transcriptome data, the miRNA-seq data, and the corresponding clinical data of BC patients were downloaded from the TCGA data portal (https://portal.gdc.cancer.gov/) for subsequent difference and co-expression analysis. It excluded patients without survival information for further evaluation. The prognostic value of miRNA and m6A RNA methylation modulators was verified in TCGA, GEO (https://www.ncbi.nlm.nih.gov/geo/), and Tang_2018 databases (http://kmplot.com/analysis/index.php?p=background). The expression profiling of circRNAs data was extracted from GSE101123.

### Identification of DE genes

From previous studies, a total of 21 m6A methylation regulators were selected for identification [3, 13], and 17 m6A methylation regulators between 1065 BC patients and 112 normal samples were finally confirmed by Mann–Whitney-Wilcoxon Test according to the available mRNA expression data from TCGA. The prognostic value of the m6A RNA methylation modulators was further assessed by univariate Cox regression survival analyses and Lasso Cox regression analysis, and those DE modulators were identified as components of the following network construction. The R package “Bioconductor Limma” was used to screen out DE miRNAs between 1057 BC and 103 normal samples in TCGA. Benjamini Hochberg method was used to calculate the adjusted P value (false discovery rate, FDR) of each gene and the threshold for DE miRNAs selection was set to FDR < 0.05 and |log_2_FC|> 1. The DE circRNAs between 8 BC and 3 normal cases were evaluated by a rank aggregation method in GSE101123. Both DE miRNAs and circRNAs were further visualized via heatmap.

### Construction of circRNA-miRNA-m6A RNA methylation modulator regulatory network

MiRDIP (http://ophid.utoronto.ca/mirDIP/) and Circular RNA Interactome (https://circinteractome.nia.nih.gov/) were used respectively to predict miRNA-m6A RNA methylation modulators interactive pairs and circRNA-miRNA interactive pairs. The miRNA-m6A modulators interactive pairs were selected after taking the intersection between the potential miRNAs targeting m6A RNA methylation regulators with the very high score (top 1%) in miRDIP and the miRNAs DE in BC tissues from TCGA project. Finally, a circRNA-miRNA-mRNA regulatory network was further constructed by taking the intersection of miRNA-m6A modulators interactive pairs and circRNA-miRNA interactive pairs.

### Gene set enrichment analysis (GSEA), the Kyoto Encyclopedia of Genes and Genomes (KEGG) and single-sample gene set enrichment analysis (ssGSEA)

The 1178 BC samples from TCGA were divided into HNRNBC high- and low-expression groups respectively, which were further analyzed by GSEA (http://software.broadinstitute.org/gsea/index.jsp) to compare the potential biological pathways and illuminate the potential regulatory mechanisms. The gene set list c5.go.bp.v7.4.symbols.gmt, c5.go.cc.v7.4.symbols.gmt, c5.go.mf.v7.4.symbols.gmt, c2.cp.kegg.v7.4.symbols.gmt were utilized as the reference gene set. The threshold was defined as nominal P < 0.05. According to the existing literature reports [14–16], some biological processes and signaling pathways closely related to BC were selected for visualization. R software (4.0.1 version) was used for KEGG pathway enrichment analyses to investigate potential differential pathways between HNRNPC high and low expression groups. To evaluate the potential immune regulatory role of HNRNPC in the BC immune infiltrations and functions, the “gsva” package was used to perform the ssGSEA to calculate the scores of infiltrating immune cells and to evaluate the activity of immune-related pathways.

### Cell culture and transfection

The human BC cell lines, MCF-7 and MDA-MB-231 were obtained from American Type Culture Collection (Manassas, VA, USA) and cultured in Dulbecco’s Modified Eagle’s Medium (DMEM) supplemented with 10% (v/v) fetal bovine serum (Gibco) at 5% CO_2_ at 37 °C. HNRNPC siRNA, hsa-miR-944 inhibitors, circBACH2 siRNA, and their corresponding negative control were purchased from Ribobio (Wuhan, China). The cells in 6 well plates were transfected with 50 nM inhibitors or siRNA by using Lipofectamine 3000 reagent (Invitrogen) according to the manufacturer’s instructions. The specific siRNA sequences for HNRNPC were provided in Additional file 1: Table S1. Three independent experiments were performed for cell transfection.

### Cell proliferation assay

Cell proliferation assay was measured using the Cell Counting Kit-8 (CCK-8) (Dojindo, Kumamoto, Japan) and 5-ethynyl-2′-deoxyuridine (EdU) incorporation assay (RiboBio, Wuhan, China) kits. In the CCK-8 assay, the transfected cells were seeded in 96-well plates at a density of 2 × 10^3^/well. Cell viability was detected from 12 to 72 h by directly adding CCK-8 reagent to each well. Finally, the optical density (OD) was recorded at a wavelength of 450 nm by a microplate reader (BioTek Instruments, United States). In the EdU assay, the BC cells were incubated with a medium containing 50 µM EdU for 2 h. After being fixed in 4% paraformaldehyde for 30 min, the BC cells were stained in Apollo reaction cocktail and Hoechst 33,342 respectively. The ratio of proliferating cells (EdU positive) to the total number of cells (DAPI positive) was observed by using a fluorescence microscope (IX35, Olympus, Japan). All the results were performed in three independent experiments.

### Quantitative real-time polymerase chain reaction (qRT-PCR) analysis

TRIzol reagent kit (Invitrogen) was performed to isolate total RNA from BC cells. The concentration and purity of total RNA were evaluated using a NanoDrop 2000 spectrophotometer (Thermo Fisher Scientific, Wilmington, DE, United States). The complementary DNA (cDNA) was synthesized by using the PrimeScript RT kit (Takara, Japan) at 103 °C for 5 s, 37 °C for 10 min, and 4 °C for 15 min. The primer sequences for detection were provided in Additional file 1: Table S2. The expression level of GAPDH was used as an internal standard control. All the gene expression levels were collected and quantified using the 2^−△△Ct^ method. The results were gained from three independent experiments.

### Western blot

Total proteins were extracted using radio-immunoprecipitation assay (RIPA) (Boster, Wuhan, China) and a bicinchoninic acid (BCA) protein assay kit (Boster, Wuhan, China) was used to estimate the protein concentration. The protein extract electrophoresed in a 10% sodium dodecyl sulfate–polyacrylamide gel electrophoresis (SDS-PAGE) at 80 V for 20 min and then 120 V for 1 h and then transferred to PVDF membranes (Biosharp, Shanghai, China) at 220 mA for 60 min. After repeated washing using tris-buffered saline containing Tween 20 (TBST) and blocking with 5% bull serum albumin (BSA) blocking buffer for 2 h at 37 °C, the PVDF membranes were incubated with primary antibodies anti-HNRNPC (1:4000), anti-GAPDH (1:5000), p-ErK(1:1500), t-Erk (1:1500), t-MAPK (1:1000) and p-MAPK (1:1500) overnight at 4 °C and then incubated with the secondary antibody of HRP-linked antibody (1:5,000, Abcam, Cambridge, MA, United States) for 1 h at 37 °C. Finally, the blots were visualized using the enhanced chemiluminescence (ECL) detection kit (Yeasen, Shanghai, China), and the relative protein abundance was measured by ImageJ image analysis software (version 1.44p, National Institutes of Health, United States). All primary antibodies were purchased from Proteintech, Wuhan, China.

### Statistical analysis

All statistical analysis was carried out by using R version 4.0.5 and GraphPad Prism 8.0. Mann–Whitney U test was used to compare the expression differences of the m6A RNA methylation regulators were compared between tumor tissues and normal tissues. The differences between the two subgroups were assessed via Student’s t-test. The independent prognostic value and other clinical characteristics were estimated by univariate and multivariate Cox proportional hazard regression analyses. Pearson correlation analyses were performed to evaluate the associations between two variables. P < 0.05 was thought to be statistically significant.

## Results

### Expression levels of m6A RNA methylation regulators were up-regulated in BC

**Fig. 1:**
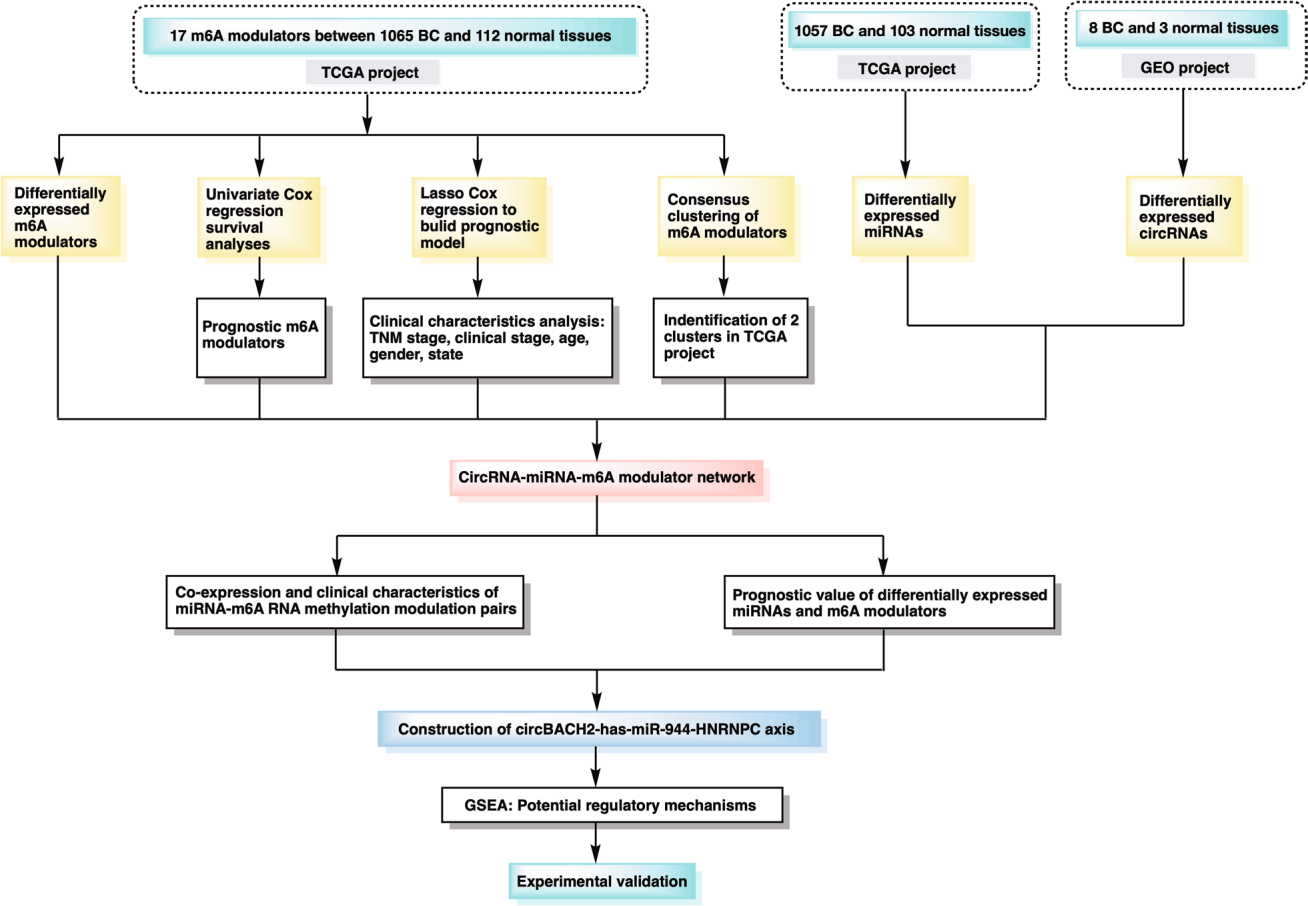
The flowchart of the study

**Fig. 2:**
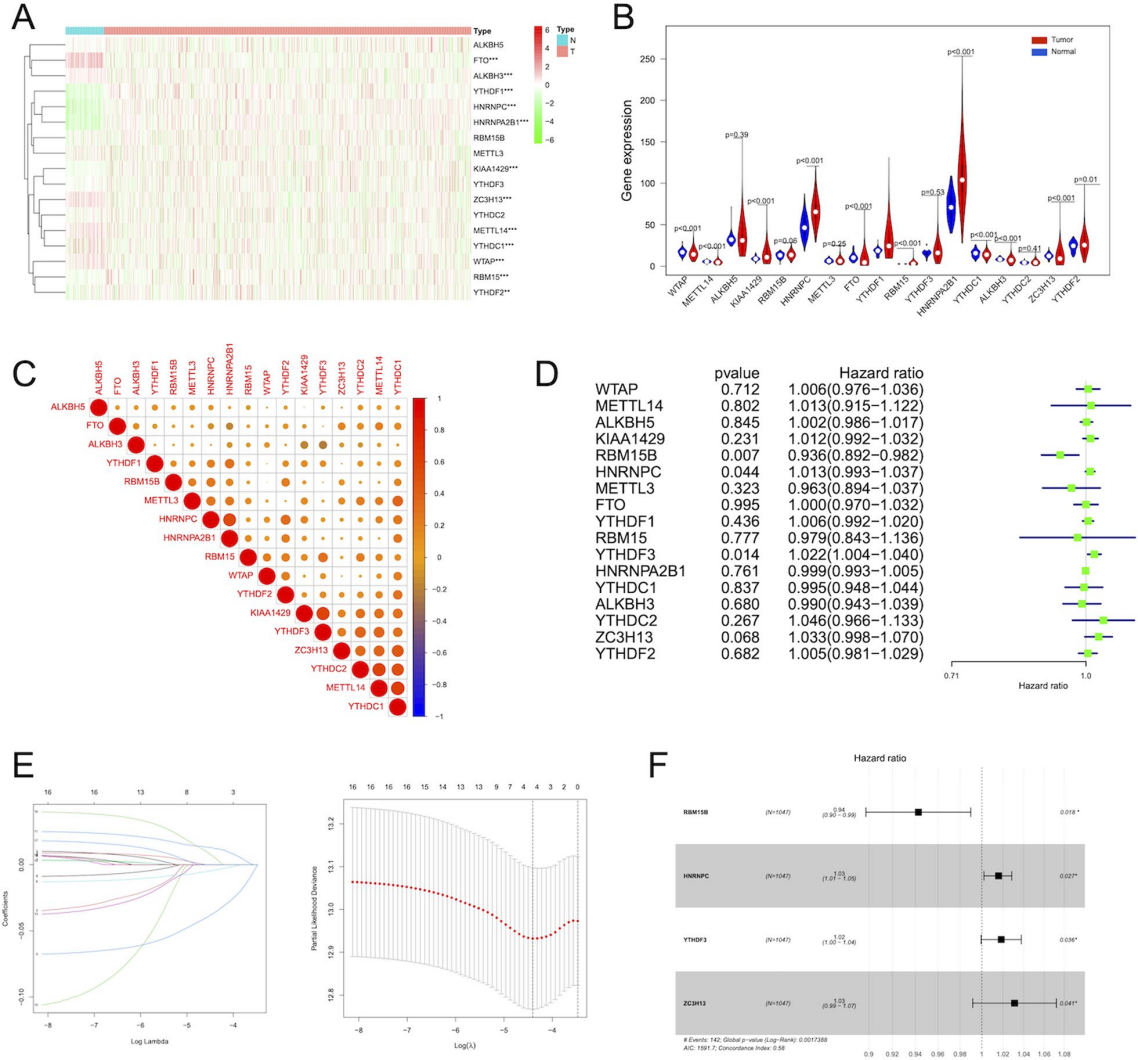
The expression and prognostic value of m6A modulators and ﻿risk model based on m6A-related genes. **A** Heatmap of the 17 prognostic m6A regulator signatures in the TCGA project. The asterisks represented the statistical P value (**P < 0.01; ***P < 0.001). **B** The expression of 17 m6A regulators between normal tissues and BC tissues from TCGA. Tumor, red; Normal, blue. **C** The Pearson correlation analysis of the relationship among m6A regulators. **D** The univariate Cox regression analysis of m6A RNA methylation modulators for OS of BC cases from TCGA. **E** Lasso Cox regression analysis of 17 m6A-related genes. **F** The forest plot of the hazard ratio for the association between 4 DE m6A modulators and OS of BC patients

The flowchart was presented in Fig. 1. In the TCGA project, after systematic investigation on the expression levels of 17 m6A regulatory genes between 1065 BC and 112 normal samples, 12 genes were significantly up-regulated in BC, while the expression levels of other 5 genes showed no significant differences (Fig. 2A, B). These results revealed that m6A regulatory genes indeed exhibited differential expression patterns between BC and normal patients, which might provide a potential tool for BC diagnosis. The heatmap visualized the correlations among those 17 m6A regulators (Fig. 2C). Then, we analyzed the prognostic values of m6A RNA methylation modulators in TCGA. To make the prognostic prediction ability of m6A modulators more credible, the clinical characteristics of the TCGA-BC data set were used as an external validation set. Additional file 1: Table S3 showed the clinical features of enrolled BC patients. The result from univariate Cox regression survival analysis indicated that RBM15B was a protective factor for prolonging the survival of BC patients, while HNRNPC and YTHDF3 were associated with poor survival (Fig. 2D). Then the Lasso and stepwise multivariate Cox regression analysis was executed to further build a signature for predicting BC prognosis, which further indicated that 4 m6A modulators including RBM15B, HNRNPC, YTHDF3, and ZC3H13 could be used to build the risk model (Fig. 2E, F). Furthermore, the data from the forest plot with the hazard ratio for correlation between the above 4 genes and OS of BC patients indicated that the higher levels of HNRNPC, YTHDF3 and ZC3H13 in BC patients were associated with the unfavorable outcome of survival (Fig. 2F). Thus, our following study mainly focused on the commonly risk m6A RNA methylation modulators with prognostic value in BC, namely HNRNPC and YTHDF3.

### The circRNA-miRNA-m6A RNA methylation modulator regulatory network in BC

**Fig. 3:**
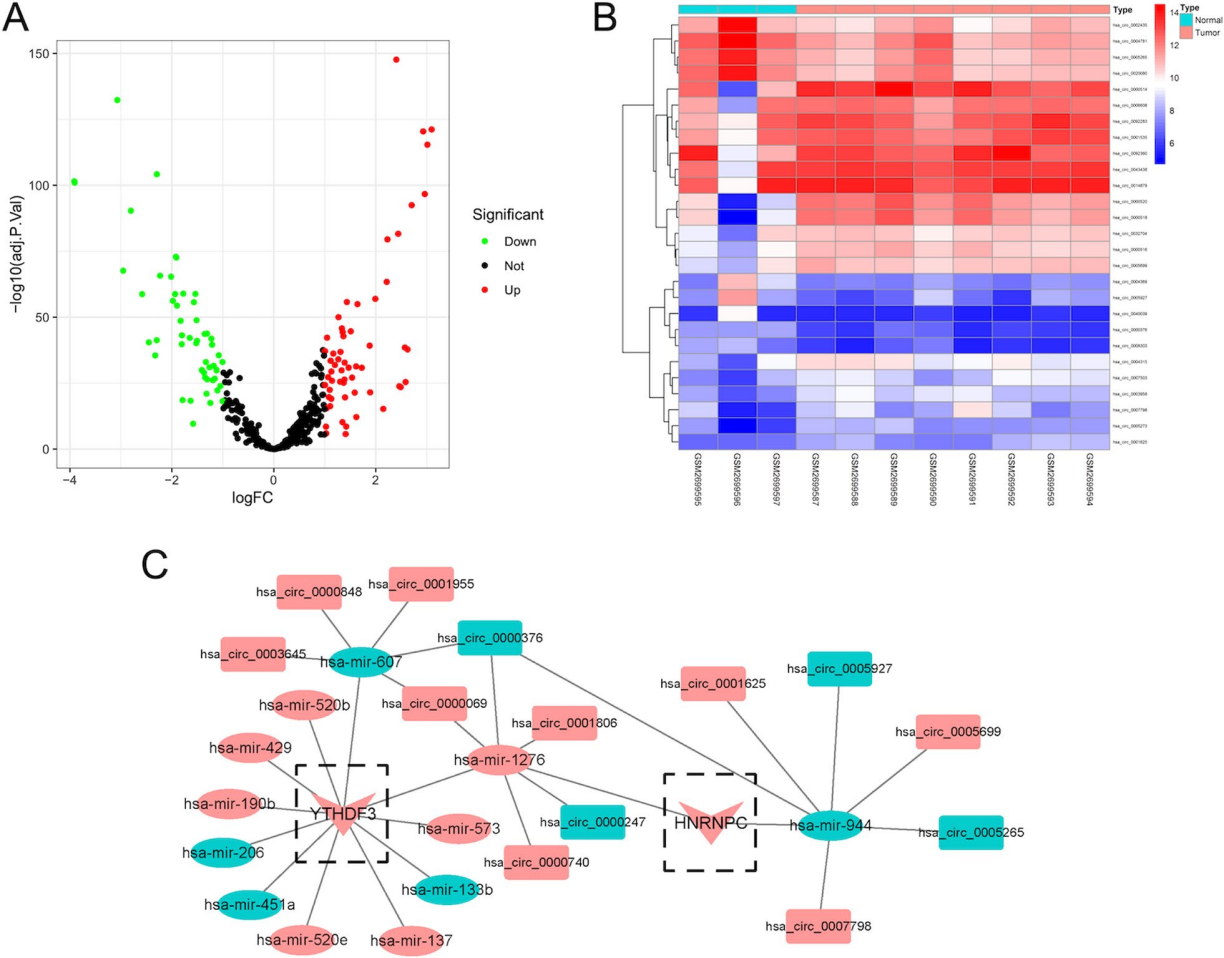
Identification of DE miRNAs and circRNAs and network construction. **A** The volcano map of 377 DE miRNAs in the TCGA project. **B** The heatmap of 27 significant DE circRNAs in GSE101123. **C** Construction of circRNA-miRNA-mRNA network. The rectangular, oval and triangle represented circRNA, miRNA, and mRNA, respectively. Red and green represented upregulation and downregulation, respectively

Next, a total of 154 DE circRNAs (96 up- and 58 down-regulation) **(**Additional file 1: Fig. S1) and a total of 377 DE miRNAs (268 up- and 109 down-regulation) (Fig. 3A) were verified between BC and normal tissues in GSE101123 and TCGA BC cases, respectively. Furthermore, the most significant 27 DE circRNAs (34 up- and 18 down-regulation) (Fig. 3B) and 30 DE miRNAs (9 up- and 21 down-regulation) (Additional file 1: Fig. S2) were selected to be visualized via the heatmap. Next, after predicting 271 miRNA-m6A RNA methylation modulators pairs and 1680 circRNA-miRNA pairs and taking the intersection of these RNA pairs, we utilized 2 m6A RNA methylation modulators (HNRNPC and YTHDF3), 12 DE miRNAs, and 11 DE circRNAs to further construct a circRNA-miRNA-m6A RNA methylation modulator regulatory network, composing of 16 circRNA-miRNA pairs and 13 miRNA-mRNA pairs (Fig. 3C).

### Co-expression and clinical characteristics of miRNA-m6A RNA methylation modulators pairs

**Fig. 4:**
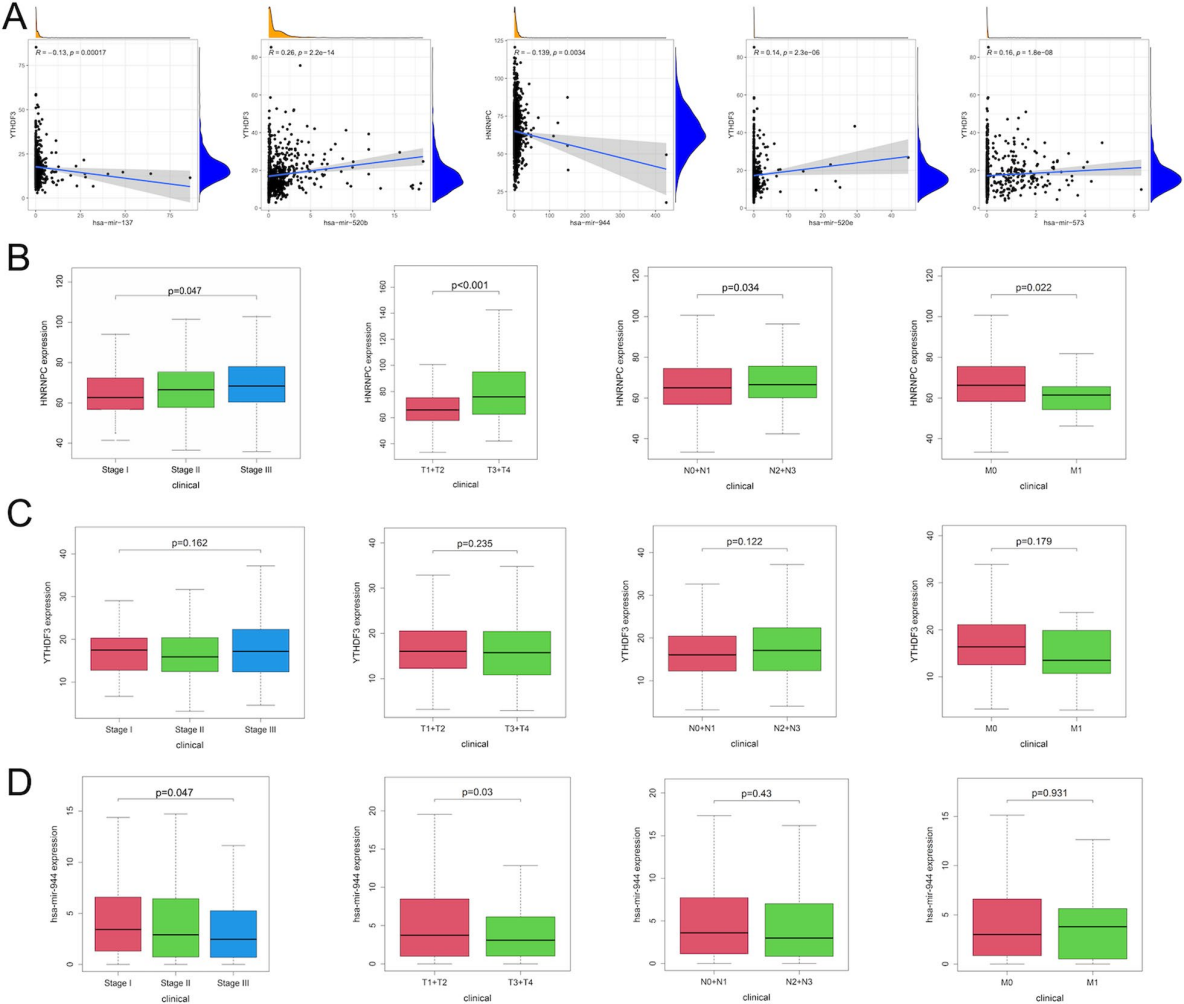
The co-expression patterns and the correlation with clinical characteristics between miRNAs and m6A modulators in the TCGA project. **A** Co-expression analysis between 5 miRNAs and 2 m6A modulators. Correlation of the expression level of HNRNPC (**B**), YTHDF3 (**C**) and hsa-miR-944 (**D**) with clinical stages and TNM stages in BC patients

Next, to confirm the most promising interactive miRNA-mRNA pairs, we analyzed expression patterns between 5 DE miRNAs and 2 m6A RNA methylation modulators via Pearson correlation analysis. As shown in Fig. 4A, 5 co-expressed miRNA-m6ARNA methylation modulator pairs were identified and hsa-miR-944 expression was significantly negatively correlated with HNRNPC (r = − 0.139, P < 0.001). Notably, hsa-miR-944 was down-regulated in BC tissues compared with normal tissues from 3 GEO datasets (Additional file 1: Table S4) and HNRNPC was up-regulated in BC tissues from other 4 GEO datasets (Additional file 1: Table S5), validating the potential negative correlation between hsa-miR-944 and HNRNPC. Subsequently, we analyzed the correlation of 2 m6A RNA methylation modulators and 5 DE miRNAs with clinical BC stages and TNM stages. Intriguingly, the high expression of HNRNPC was significantly correlated with late clinical-stage III, and late T stage and N stage (Fig. 4B). However, there exhibited no significant correlations of the expression of YTHDF3 with clinical BC stages and TNM stages (Fig. 4C). In addition, after further investigating the correlation of 5 DE miRNAs with BC clinical characteristics, it was interesting to find that only the high expression of hsa-miR-944 was significantly correlated with the early clinical-stage I and T stage in BC (P < 0.05), while the expression of hsa-miR-944 displayed no statistically correlation with N stage and M stage (Fig. 4D, Additional file 1: Fig. S3). Thus, it proposed the hsa-miR-944-HNRNPC pair as the most interactive pairs based on the clinical significance.

### Prognostic value of hsa-miR-944 and HNRNPC and construction of circRNA-hsa-miR-944-HNRNPC network

**Fig. 5:**
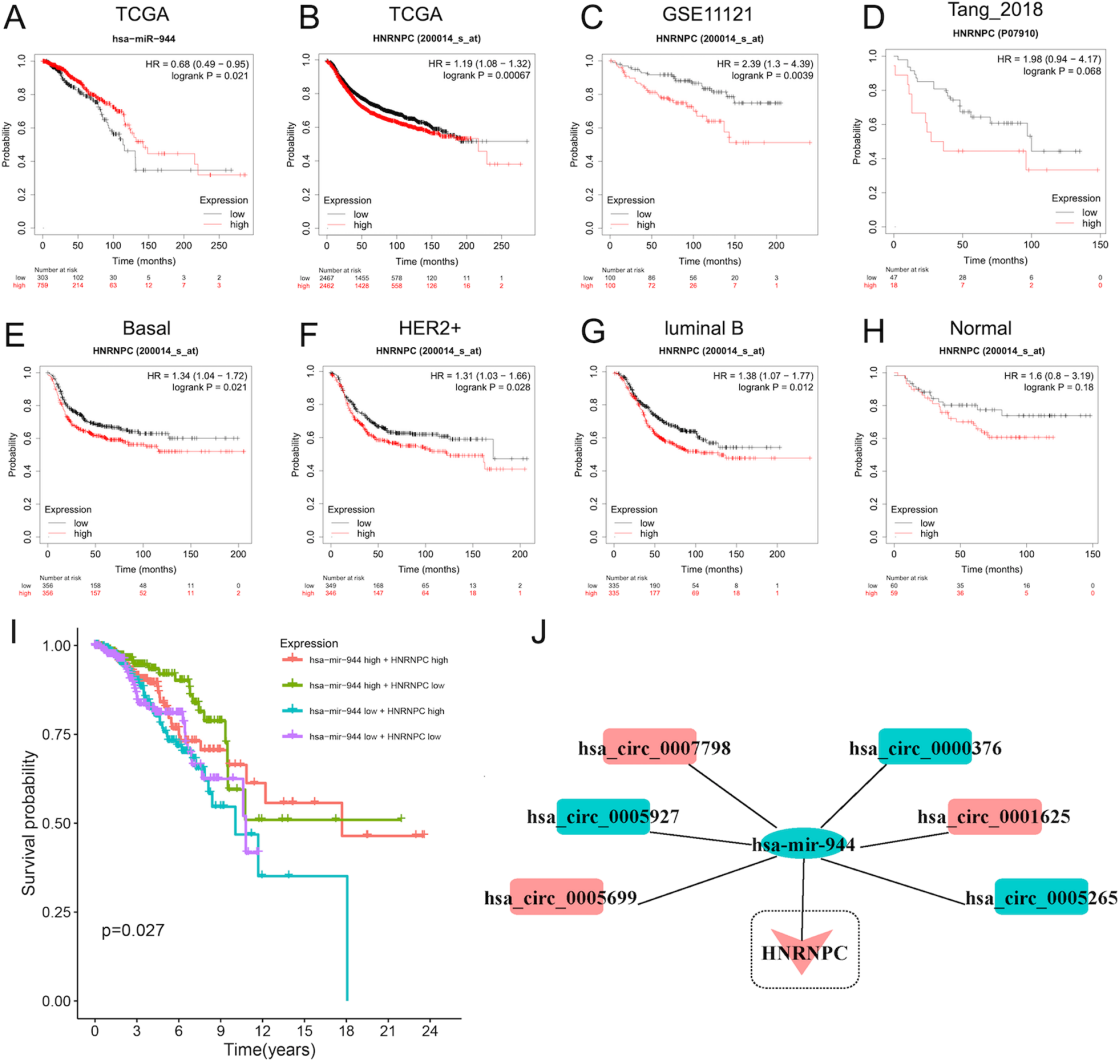
﻿The prognostic value of HNRNPC and hsa-miR-944 and construction of the hub circRNA-hsa-miR-944-HNRNPC regulatory network in BC. **A** The Kaplan–Meier survival analysis about BC patients with hsa-miR-944 high and low expression level. The Kaplan–Meier survival analysis about BC patients with HNRNPC high and low expression level in TCGA project (**B**), GSE11121 database (**C**) and Tang_2018 database (**D**). The Kaplan–Meier survival analysis about Basal-like patients (**E**), HER2 + patients (**F**), Luminal B-like patients (**G**), and normal-like patients (**H**) with HNRNPC high and low expression level. **I** The Kaplan–Meier survival analysis of BC patients with different expression level of hsa-miR-944 and HNRNPC in TCGA project. **J** The construction of hub circRNA-hsa-miR-944-HNRNPC regulatory network. The rectangular, oval and triangle represented circRNA, miRNA, and mRNA, respectively. Red and green represented upregulation and downregulation, respectively

The survival analysis verified that the high expression of hsa-miR-944 predicted good prognosis and long-time survival of BC patients (P < 0.05) (Fig. 5A), while the high expression of HNRNPC predicted shorter OS of BC patients (P < 0.001) (Fig. 5B). And the prognostic value of HNRNPC had similar trends in GSE11121 (Fig. 5C), and Tang_2018 databases (Fig. 5D), which were consistent with the TCGA project. Additionally, given the high heterogeneity of BC, we further identify the prognostic value of HNRNPC in 5 BC subtypes. It found that except for luminal A (P > 0.05) (Additional file 1: Fig. S4), the high expression of HNRNPC was associated with poor prognosis of basal-like, HER2 + , luminal B and normal-like BC patients (P < 0.05) (Fig. 5E–H). Therefore, according to the co-expressed patterns of miRNA-m6ARNA methylation modulator pairs, it was speculated that the low expression of hsa-miR-944 and high expression of HNRNPC were associated with unsupported progression of BC. As expected, the results validated that BC patients with hsa-miR-944 low and HNRNPC high expression had shorter OS time than those with contrast expression level (Fig. 5I). Moreover, only the OS of the basal-like and luminal B-like BC patients with high expression of HNRNPC and low expression of has-mir-944 was shorter than other groups (Additional file 1: Fig. S5). Hence, the circRNA-miRNA-mRNA regulatory network with prognostic value in BC could be constructed based on HNRNPC and hsa-miR-944, which consisted of a total of 6 regulatory axes, including hsa_circ_0000376/hsa-miR-944/HNRNPC, hsa_circ_0001625/hsa-miR-944/HNRNPC, hsa_circ_0005927/hsa-miR-944/HNRNPC, hsa_circ_0005699/hsa-miR-944/HNRNPC, hsa_circ_0005265/hsa-miR-944/HNRNPC, hsa_circ_0007798/hsa-miR-944/HNRNPC (Fig. 5J).

### Potential regulatory mechanisms in DE HNRNPC groups

**Fig. 6:**
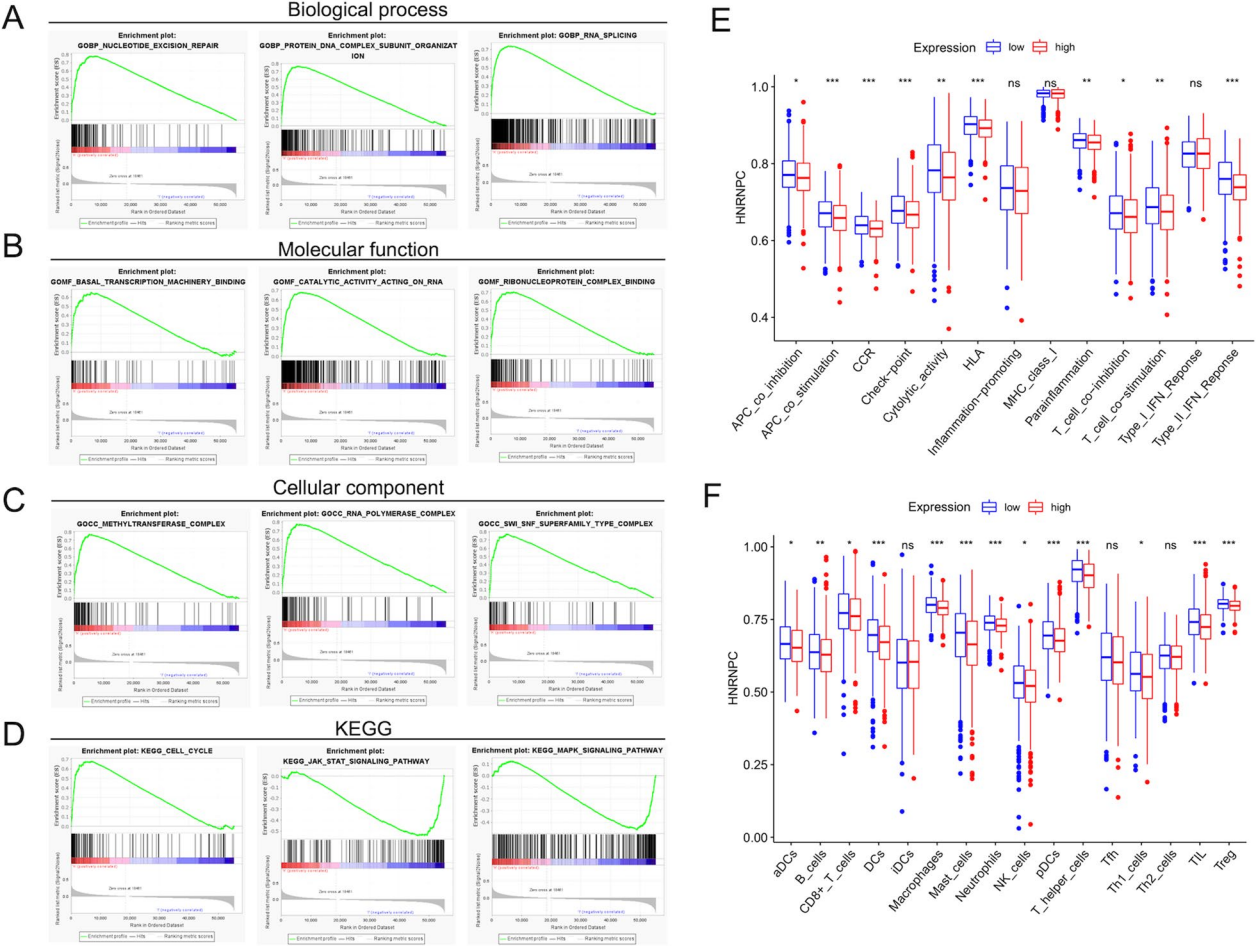
The GSEA, KEGG and ssGSEA between HNRNPC high- and low- expression groups with BC in TCGA project. The functional analysis between HNRNPC high and low expression groups, including the biological process (**A**) (nucleotide excision repair, protein-DNA complex subunit organization and RNA splicing), the molecular function (**B**) (basal transcription machinery binding, catalytic activity acting on RNA and ribonucleoprotein complex binding) and the cellular component (**C**) (methyltransferase complex, RNA polymerase complex and SWI-SNF superfamily type complex). **D** The KEGG analysis about different enriched pathways between HNRNPC high and low expression groups. The enrichment scores of 16 immune cell infiltration (**E**) and 13 immune-related functions (**F**) between HNRNPC high and low expression groups

The results from GSEA suggested that processes associated with gene expression and mRNA stability such as nucleotide excision repair, RNA splicing, and RNA polymerase complex were enriched in HNRNPC high expression group in GEO datasets, emphasizing the functional modification of m6A in eukaryotes (Fig. 6A–C). Importantly, the KEGG analysis unraveled that the relevant pathway of tumor malignancy, including JAK/STAT and MAPK signaling pathways, was significantly correlated with the HNRNPC low expression group, while the process of the cell cycle was correlated with HNRNPC high expression group (P < 0.05) (Fig. 6D). Thus, it was presumed that the discrepant expression of HNRNPC might be obsessed with the cell cycle, the JAK/STAT and the MAPK signaling pathway in BC. Furthermore, after quantifying the enrichment scores of 16 immune cell infiltration and 13 immune-related functions by using the ssGSEA analysis, we found that BC patients with high and low expression of HNRNPC displayed several specific differences of immune infiltration and functions (Fig. 6E, F), which proposed an interesting chance for us to explore the potential association of HNRNPC with tumor immunity in the future study.

### CircBACH2 promotes BC cell proliferation via hsa-miR-944/HNRNPC axis

**Fig. 7:**
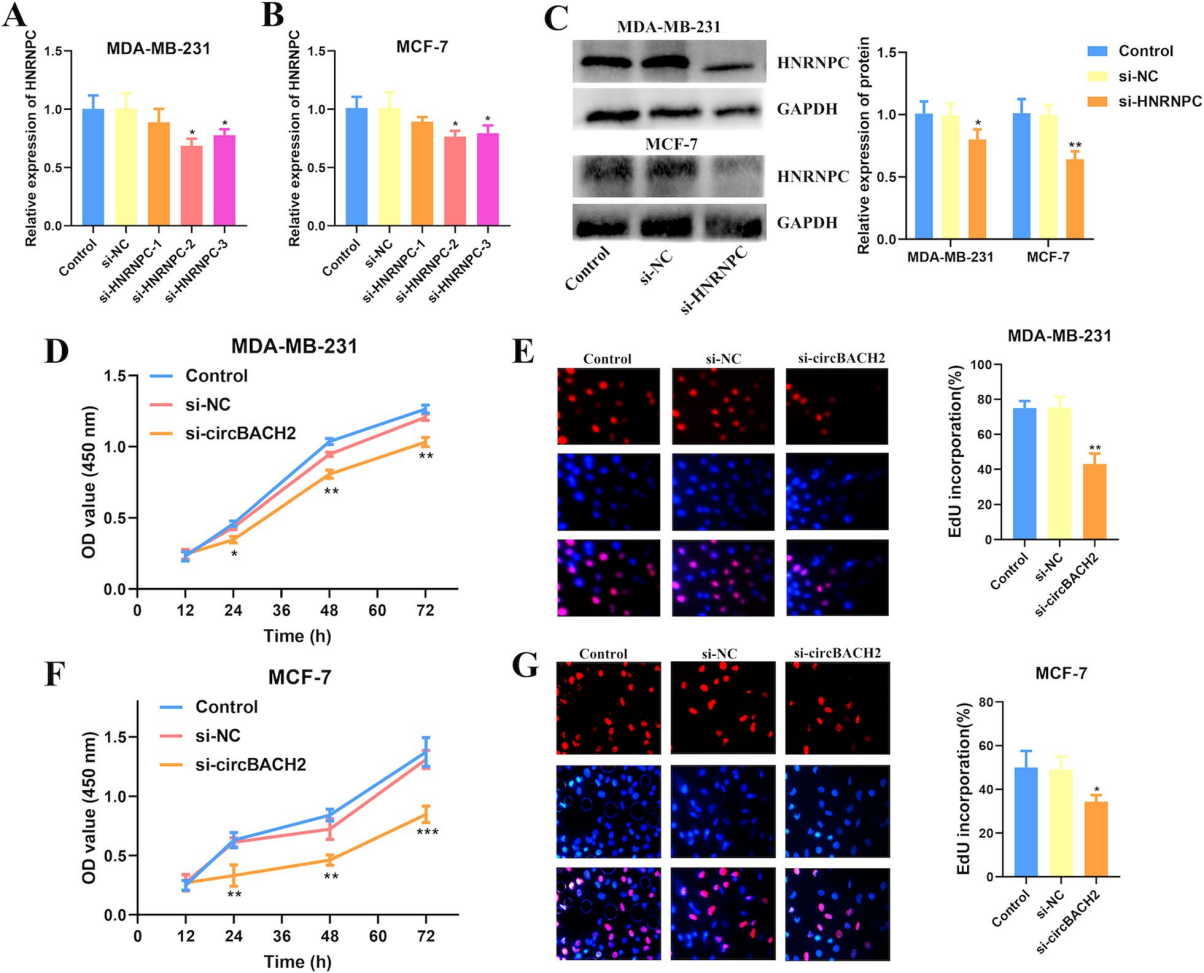
Up-expression of circBACH2 and HNRNPC promoted the proliferation of BC cells. qRT-PCR analysis of HNRNPC expression in MDA-MB-231 cell (**A**) and MCF-7 cell (**B**) after transfection with si-HNRNPC. (**C**) Western blot analysis of HNRNPC expression in MDA-MB-231 and MCF-7 cells after transfection with si-circBACH2. The CCK8 assay (**D**) and the EdU assay (**E**) indicated the proliferation of MDA-MB-231 cell after transfection with circBACH2 siRNAs or negative control. The CCK8 assay (**F**) and the EdU assay (**G**) indicated the proliferation of MCF-7 cell after transfection with circBACH2 siRNAs or negative control. *P < 0.05 vs. siRNA negative control

**Fig. 8:**
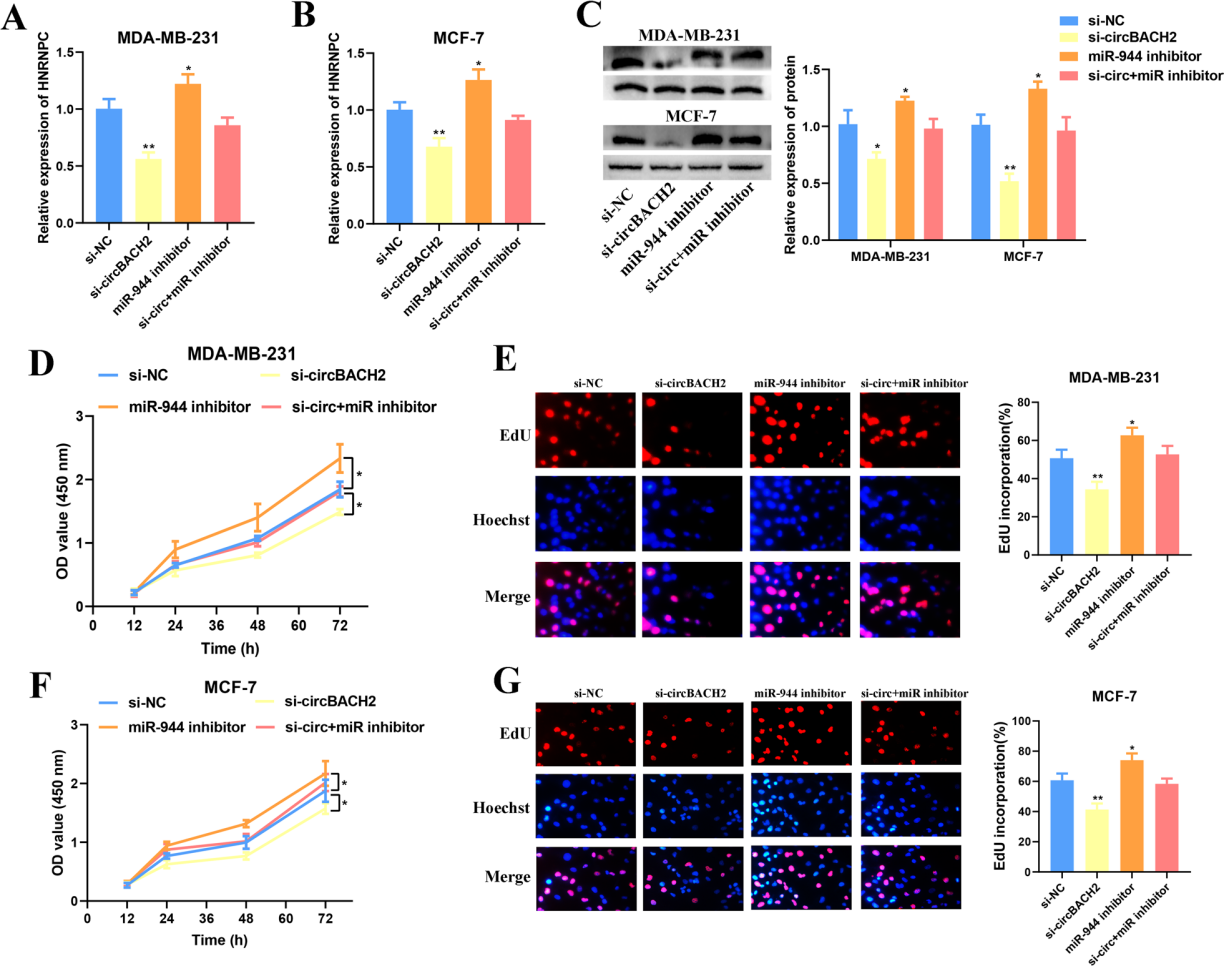
Up-expression of circBACH2 reversed the inhibitory effects of has-miR-944 on the proliferation of BC cells. qRT-PCR analysis of HNRNPC expression in MDA-MB-231 cell (**A**) and MCF-7 cell (**B**) after transfection with si-circBACH2 alone, has-miR-944 inhibitor alone and si-circBACH2 + has-miR-944 inhibitor group. **C** Western blot analysis of HNRNPC expression in MDA-MB-231 and MCF-7 cells after transfection with si-circBACH2 alone, has-miR-944 inhibitor alone and si-circBACH2 + has-miR-944 inhibitor group. The CCK8 assay (**D**) and the EdU assay (**E**) indicated the proliferation of MDA-MB-231 cells after transfection with si-circBACH2 alone, has-miR-944 inhibitor alone and si-circBACH2 + has-miR-944 inhibitor group. The CCK8 assay (**D**) and the EdU assay (**E**) indicated the proliferation of MCF-7 cells after transfection with si-circBACH2 alone, has-miR-944 inhibitor alone and si-circBACH2 + has-miR-944 inhibitor group. *P < 0.05 vs. siRNA negative control

In our study, 6 dysregulated circRNAs were identified to be involved in regulating BC progression and prognosis via hsa-miR-944/HNRNPC axis. The analytical results from the online site (https://circinteractome.nia.nih.gov/) uncovered that circBACH2 (hsa_circ_0001625) had the highest interaction score with hsa-miR-944. Moreover, circBACH2 was reported to be associated with progressions of various cancers, including TNBC [17] and papillary thyroid carcinoma (PTC) [18]. Therefore, circBACH2 was selected to be investigated for its regulatory roles in BC in our following study. Firstly, the results from cell transfection showed that the RNA and protein expression of HNRNPC could be effectively decreased by siRNA in MDA-MB-231 and MCF-7 cells (Fig. 7A–C). After transfection with HNRNPC siRNAs, the proliferation of MCF-7 and MDA-MB-231 cells were attenuated significantly by using the CCK-8 assay (Additional file 1: Fig. S6). Next, it found that the proliferation of MDA-MB-231 and MCF-7 cells was also inhibited after transfection of circBACH2 siRNAs (Fig. 7D, F). Similarly, knockdown of circBACH2 significantly decreased the percentage of EdU positive cells, suggesting that circBACH2 in itself could hinder the cell proliferative potential (Fig. 7E, G). Then, we analyzed the role of circBACH2 on modulating hsa-miR-944/HNRNPC axis. Transfection of hsa-miR-944 inhibitor significantly increased the total RNA and protein levels of HNRNPC in MDA-MB-231 and MCF-7 cells, whereas these effects were reversed by circBACH2 knockdown (Fig. 8A–C), demonstrating the potential HNRNPC modulation patterns regulated by circBACH2-hsa-miR-944 network. Additionally, the results from CCK8 and EdU assay showed that inhibition of hsa-miR-944 expression significantly promoted the proliferation of BC cells. However, circBACH2 elimination partly abrogated the stimulative effect of hsa-miR-944 on cell proliferation (Fig. 8D–G). These results concluded that circBACH2 acted as the has-miR-944 sponge to modulate the expression and activity of HNRNPC, thereby affecting the proliferation of BC cells.

### CircBACH2/ has-miR-944/HNRNPC axis regulates BC progression via the MAPK signaling pathway

**Fig. 9:**
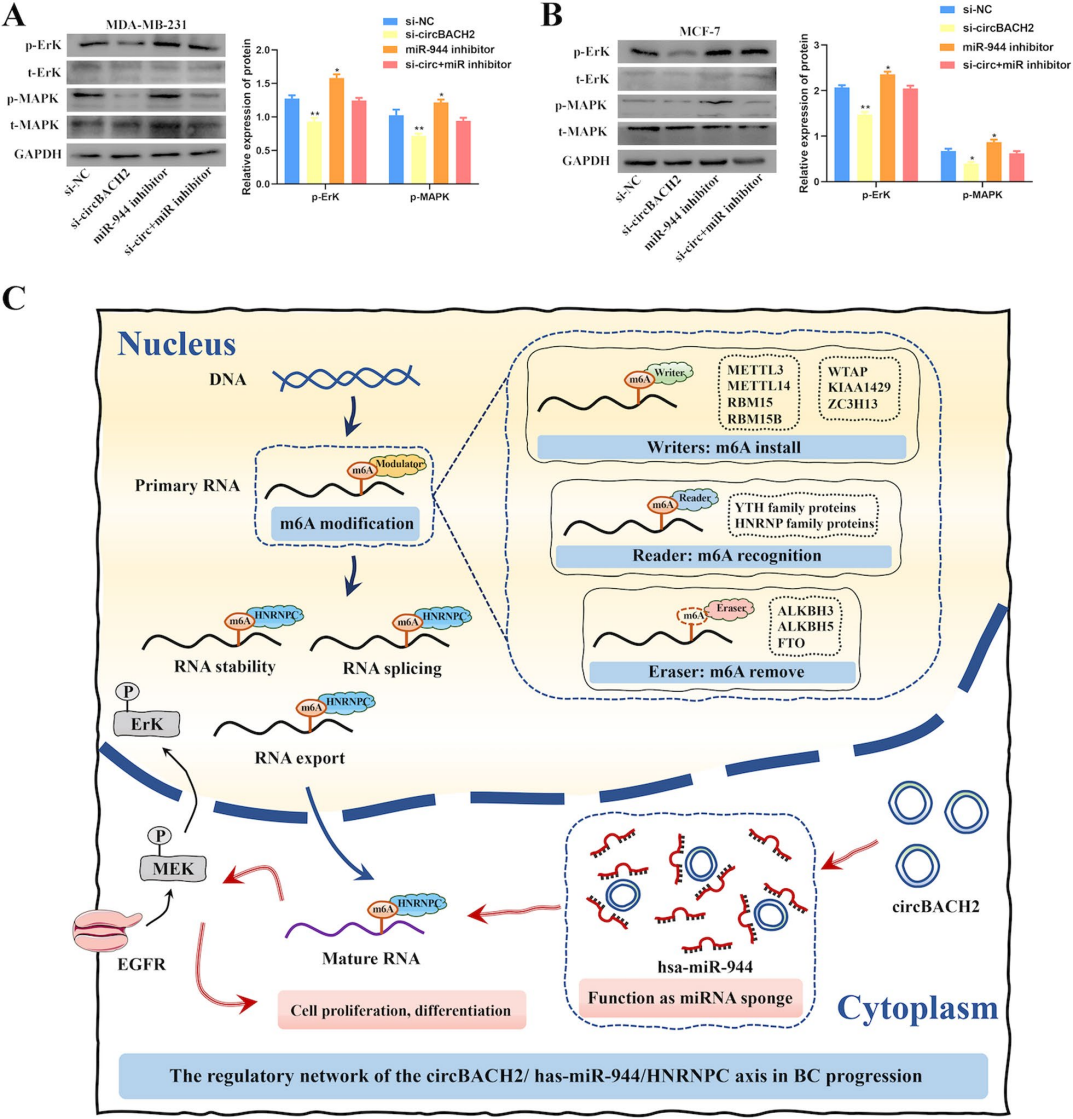
CircBACH2/ has-miR-944/HNRNPC axis regulated BC progression via the MAPK signaling pathway. Western blot analysis of the protein levels of t-ErK and t-MAPK, and the protein phosphorylation levels of p-ErK and p-MAPK in MDA-MB-231 cell (**A**) and MCF-7 cell (**B**) after transfection with si-circBACH2 alone, has-miR-944 inhibitor alone, and si-circBACH2 + has-miR-944 inhibitor group. *P < 0.05 vs. siRNA negative control. **C** The illustration of m6A modulators in m6A modification and the potential regulatory mechanisms of the circBACH2/has-miR-944/HNRNPC axis in BC proliferation and progression

A series of experiments were performed to further investigate the potential regulatory roles of circBACH2/has-miR-944/HNRNPC axis in BC progression. Western blot analysis revealed that inhibiting hsa-miR-944 expression significantly up-regulated the protein phosphorylation levels of ErK and MAPK in MDA-MB-231 and MCF-7 cells, whereas the above protein levels were down-regulated after transfection of circBACH2 siRNA. Interestingly, transfection of circBACH2 siRNA efficaciously impeded the elevated effects of hsa-miR-944 inhibition on the protein phosphorylation levels of ErK and MAPK (Fig. 9A, B). The results unveiled that there was a significantly negative correlation between circBACH2 and hsa-miR-944, as well as a highly negative correlation between hsa-miR-944 and ErK and MAPK. Taken together, it speculated that the up-regulated circBACH2 functioned as a sponge of has-miR-944 in the cytoplasm to promote the expression and activity of HNRNPC to accelerate BC cell proliferation, and that the circBACH2/ has-miR-944/HNRNPC axis regulated BC progression via the MAPK signaling pathway (Fig. 9C).

## Discussion

Tumorous cells undergo genetic and epigenetic changes to obtain malignant transformation, including the modifications of mRNA transcripts. Among them, the m6A modification emerges as one of the most abundant RNA modifications in eukaryotic cells, involving RNA processing, nuclear export, and RNA translation. There is accumulating evidence indicating that m6A methylation affects the complexity of multiple cancer progression and thus plays important role in cancers. In this study, we focused on m6A-associated mechanisms and functions in BC, and presumed and validated the potential role of the circRNA-miRNA network in regulating m6A RNA methylation modulators in BC progression. In this work, we have constructed a circRNA- and m6A- based interaction network of circBACH2/hsa-miR-944/HNRNPC axis in BC. The hsa-miR-944/HNRNPC axis was determined to be associated with clinicopathological characteristics and prognosis of BC. More importantly, circMAP2K4 could sponge the hsa-miR-944 and subsequently counteract the inhibition effect of hsa-miR-944 on HNRNPC expression, which ultimately boosts the BC proliferation and progression.

In our study, RBM15B, HNRNPC, YTHDF3 and ZC3H13 were the eventually identified modulators of m6A RNA methylation based on their differential expression and prognostic values in the TCGA projects. And finally, HNRNPC was used to construct the interacting network. It is noted that m6A RNA methylation regulators impact the BC prognosis. Wang et al. reported that the m6A methylation regulators were observably dysregulated in TNBC tissues, including up-regulated KIAA1429, YTHDF2, RBM15, and down-regulated ZC3H13, METTL14, and FTO [19]. These altered regulators constituted a meaningful prognostic signature for predicting survival of TNBC patients [20]. By bioinformatic analysis, Gong et al. showed that the expressions of METTL14 and ZC3H13 mRNA were down-regulated in BC, and indicated their synergetic roles in regulating BC cell proliferation, invasion, and metastasis [21]. As a m6A reader, YTHDF3 was overexpressed and clinically correlated with breast cancer brain metastases (BCBM), suggesting that YTHDF3 overexpression was indispensable for multiple steps of BCBM through facilitating ST6GALNAC5, GJA1, EGFR, and VEGFA expressions [22]. Anita et al. also verified that the genetic alterations of YTHDF3 were frequently associated with poor prognosis in BC patients, suggesting their transcripts upregulation might promote BC progression via a m6A-dependent manner [23]. The RNA-binding protein HNRNPC is highly expressed and can suppress the accumulation of immunostimulatory RNAs in BC cells [24]. Our results also showed that HNRNPC was a highly expressed m6A RNA methylation regulator in BC. These alterations in the m6A proteins that write, recognize or erase the m6A could lead to extensive transformation in multiple cellular processes and play critical roles in the pathogenesis of BC.

Dysfunction of miRNAs is a typical repertoire in cancer and is strongly emphasized to implicate in the pathogenesis and tumorigenicity of BC. Recently, miR-944 has been reported to possess multi-faceted characteristics in playing either oncogenic or tumor-suppressive roles in various human malignancies. For example, miR-944 may function as a tumor suppressor to inhibit colorectal cancer (CRC) by regulating GATA6, and their expression level was negatively associated with the pathological manifestation of CRC [25]. But in BC, it was reported that miR-944 was confirmed as an oncogene to mediate the chemoresistance of BC [26]. Conversely, Flores-Pérez et al. demonstrated that miR-944 was markedly suppressed in BC cell lines and tumors independent of hormonal status and stage, and could inhibit BC cell migration and invasion [26]. Whereas in our study, miR-944 emerged as a cancer suppressor gene to restrain the HNRNPC. This result is consistent with the study of Flores-Pérez. Due to the limited studies, the underlying roles and mechanisms of miR-944 on BC need to be further confirmed.

CircRNAs are ubiquitous non-coding RNAs in eukaryotic cells, which are diverse, stable, and evolutionarily conserved. It is gradually recognized that circRNA is dysregulated in BC tissues and participated in the pathogenesis of BC by harboring miRNAs [27]. For instance, Yang et al. successfully identified a total of 47 upregulated and 307 downregulated DE circRNAs to construct the competing endogenous RNA (ceRNA) network of BC [28]. Additionally, the upregulated circAGFG1 was associated with accelerated cell division and poor prognosis involving the circRNA-miRNA-hub gene network in the pathogenesis of BC.

However, although in the past few years, many studies have attempted to decipher the properties of expression abundance, functions, and mechanism of m6A RNA methylation regulators and their interaction with miRNAs in BC, the interaction of circRNA involved in the interaction of miRNAs and modulators are remain not very clear. Besides, few studies have reported the function of the circRNA-miRNAs-m6A axis in BC, regardless of BC stage or type. This inspired us to explore the potential correlation between the circRNA and m6A modification. CircBACH2 was the screened and selected circRNA target, which was proved to be up-regulated in TNBC cancerous tissues and was associated with malignant progression in patients with TNBC [17]. Mechanistically, the abnormally expressed circBACH2 acted as an oncogenic circRNA in TNBC via miR-186-5p and miR-548c-3p/CXCR4 axis. Interestingly, Cai et al. also investigated the function of circBACH2 in papillary PTC, and found that circBACH2 could bind to miR-139-5p to serve as a pro-tumorigenic RNA through a circBACH2/miR-139-5p/LMO4 axis in PTC [18]. Here, we confirmed that circBACH2 was able to promote BC cell proliferation by combining with hsa-miR-944 to promote HNRNPC expression, thus establishing a circRNA-miRNA-mRNA regulatory network in BC. Meanwhile, our results were also consistent with previous reports that circBACH2 functioned as an oncogene in BC development. Besides, the MAPK signaling pathway is known to regulate various cellular activities related to cancer progression including proliferation, differentiation, apoptosis, and immune escape [29]. According to relevant literature reports, the BC cell stemness and metastasis could be increased via activating the MAPK pathway [30, 31]. Notably, non-coding RNAs have emerged as potential vital regulators in BC progression. For example, miR-188 inhibited BC cell migration and promoted apoptosis by suppressing the activation of the MAPK signaling pathway to negatively regulate Rap2c [32]. Therefore, the MAPK/ERK pathway was selected to investigate how the circBACH2/hsa-miR-944/HNRNPC axis promoted BC cell proliferation. Western blot analysis suggested that the protein phosphorylation levels of ErK and MAPK in BC cells were increased after transfection with hsa-miR-944 inhibitors, while the above stimulative effects could be reversed by circBACH2 siRNAs. The results unveiled that the overexpression of circBACH2 could promote phosphorylation of the MAPK signaling pathway. Thus, we propose that circBACH2 mediated stimulation of BC cell proliferation via activating the MAPK signaling pathway by acting as the hsa-miR-944 sponge to promote HNRNPC expression. All of these results uncovered a novel tumor-boosting mechanism in BC, indicating that the circBACH2/hsa-miR-944/HNRNPC axis may serve as a promising therapeutic target in BC patients.

## Conclusions

Overall, the current study revealed that circBACH2 sponged hsa-miR-944 to up-regulate the expression of m6A RNA methylation modulator HNRNPC, thus accelerating the progression of BC. It successfully defined that circBACH2 functioned as an oncogenic circRNA via the circBACH2/hsa-miR-944/HNRNPC axis, which is hoping to provide a novel diagnostic and therapeutic target for BC.

## Supplementary Information

**Additional file 1:** **Figure S1.** The volcano map of 154 DE circRNAs of BC patients from GSE101123. **Figure S2.** Heatmap of 30 DE miRNAs of BC patients from TCGA project. **Figure S3.** The correlation of the other 4 DE miRNAs with BC clinical stages and TNM stages. **Figure S4.** The Kaplan-Meier survival analysis about Luminal A-like patients with hsa-miR-944 high and low expression level. **Figure S5.** The Kaplan-Meier survival analysis of 5 subtype BC patients with the different expression levels of hsa-miR-944 and HNRNPC. **Figure S6.** The CCK8 assay indicated the proliferation of MCF-7 cells (A) and MDA-MB-231 cells (B) after transfection with HNRNPC siRNAs or negative control. **Table S1.** siRNA sequences for HNRNPC. **Table S2.** Primers for qRT-PCR. **Table S3.** Summary of clinical characteristics of TCGA-BC dataset. **Table S4.** The expression of 5 differentially expressed miRNAs in different GEO datasets. **Table S5.** The expression of 2 differentially expressed mRNAs in different GEO datasets.

## Data Availability

The datasets presented in this study could be found in online database. The original contributions presented in the study are deposited in the article and Supplementary Material, further inquiries can be directed to the corresponding author.

## Abbreviations

EdU: 5-Ethynyl-2′-deoxyuridine
BCA: Bicinchoninic acid
BC: Breast cancer
BCBM: Breast cancer brain metastases
BSA: Bull serum albumin
CCK-8: Cell Counting Kit-8
circRNAs: Circular RNAs
CRC: Colorectal cancer
ceRNA: Competing endogenous RNA
CDF: Cumulative distribution function
DE: Differentially expressed
DMEM: Dulbecco’s Modified Eagle’s Medium
ECL: Enhanced chemiluminescence
GEO: Gene Expression Omnibus
GSEA: Gene set enrichment analysis
miRNA: MicroRNA
m6A: N6-methyladenosine
OD: Optical density
OS: Overall survival
qRT-PCR: Quantitative real time polymerase chain reaction
RIPA: Radio-immunoprecipitation assay
TCGA: The Cancer Genome Atlas
PTC: Thyroid carcinoma
TNBC: Triple-negative breast cancer
TBST: Tris-buffered saline containing Tween
