# Supplementary material for: Analysis and validation of m6A regulatory network: a novel circBACH2/has-miR-944/HNRNPC axis in breast cancer progression
Source: J Transl Med. 2021 Dec 24;19:527. doi: 10.1186/s12967-021-03196-4 (PMC8709995; doi:10.1186/s12967-021-03196-4)
Supplement: Supplementary file 1 — Additional file 1: Figure S1. The volcano map of 154 DE circRNAs of BC patients from GSE101123. Figure S2. Heatmap of 30 DE miRNAs of BC patients from TCGA project. Figure S3. The correlation of the other 4 DE miRNAs with BC clinical stages and TNM stages. Figure S4. The Kaplan-Meier survival analysis about Luminal A-like patients with hsa-miR-944 high and low expression level. Figure S5. The Kaplan-Meier survival analysis of 5 subtype BC patients with the different expression levels of hsa-miR-944 and HNRNPC. Figure S6. The CCK8 assay indicated the proliferation of MCF-7 cells (A) and MDA-MB-231 cells (B) after transfection with HNRNPC siRNAs or negative control. Table S1. siRNA sequences for HNRNPC. Table S2. Primers for qRT-PCR. Table S3. Summary of clinical characteristics of TCGA-BC dataset. Table S4. The expression of 5 differentially expressed miRNAs in different GEO datasets. Table S5. The expression of 2 differentially expressed mRNAs in different GEO datasets. [file 12967_2021_3196_MOESM1_ESM.docx]

# Supplementary Material

## 1. Supplementary Figures

**Figure S1**. The volcano map of 154 DE circRNAs of BC patients from GSE101123.

**Figure S2.** Heatmap of 30 DE miRNAs of BC patients from TCGA project.

**
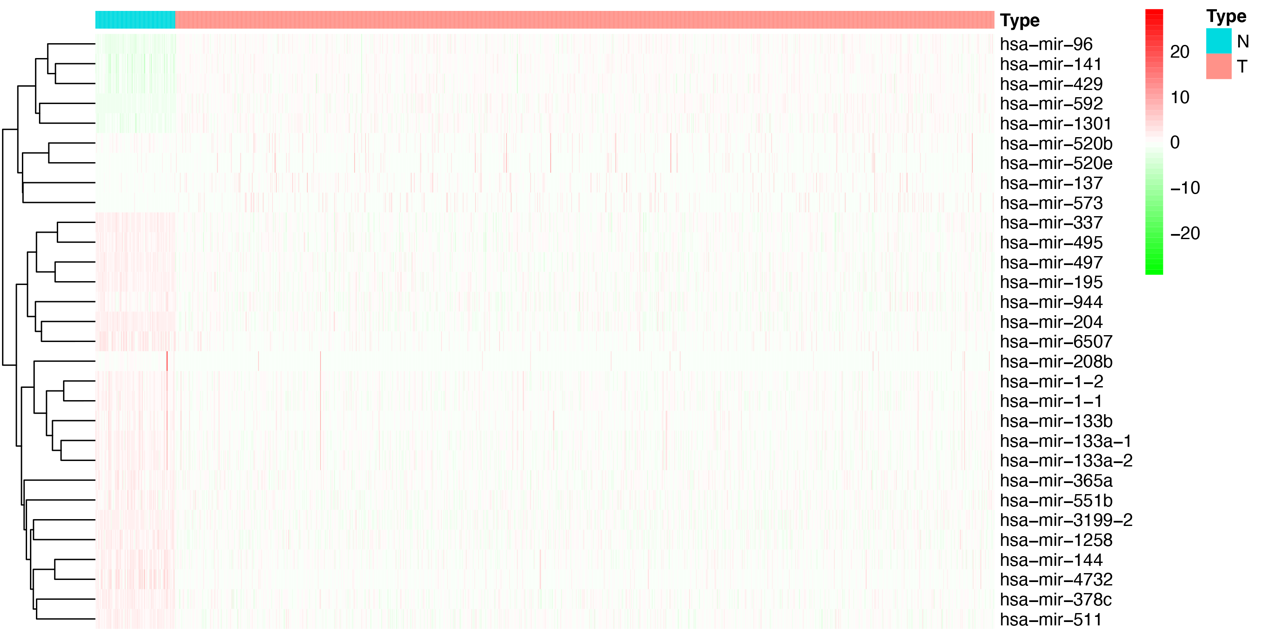
**

**Figure S3.** The correlation of the other 4 DE miRNAs with BC clinical stages and TNM stages.

**
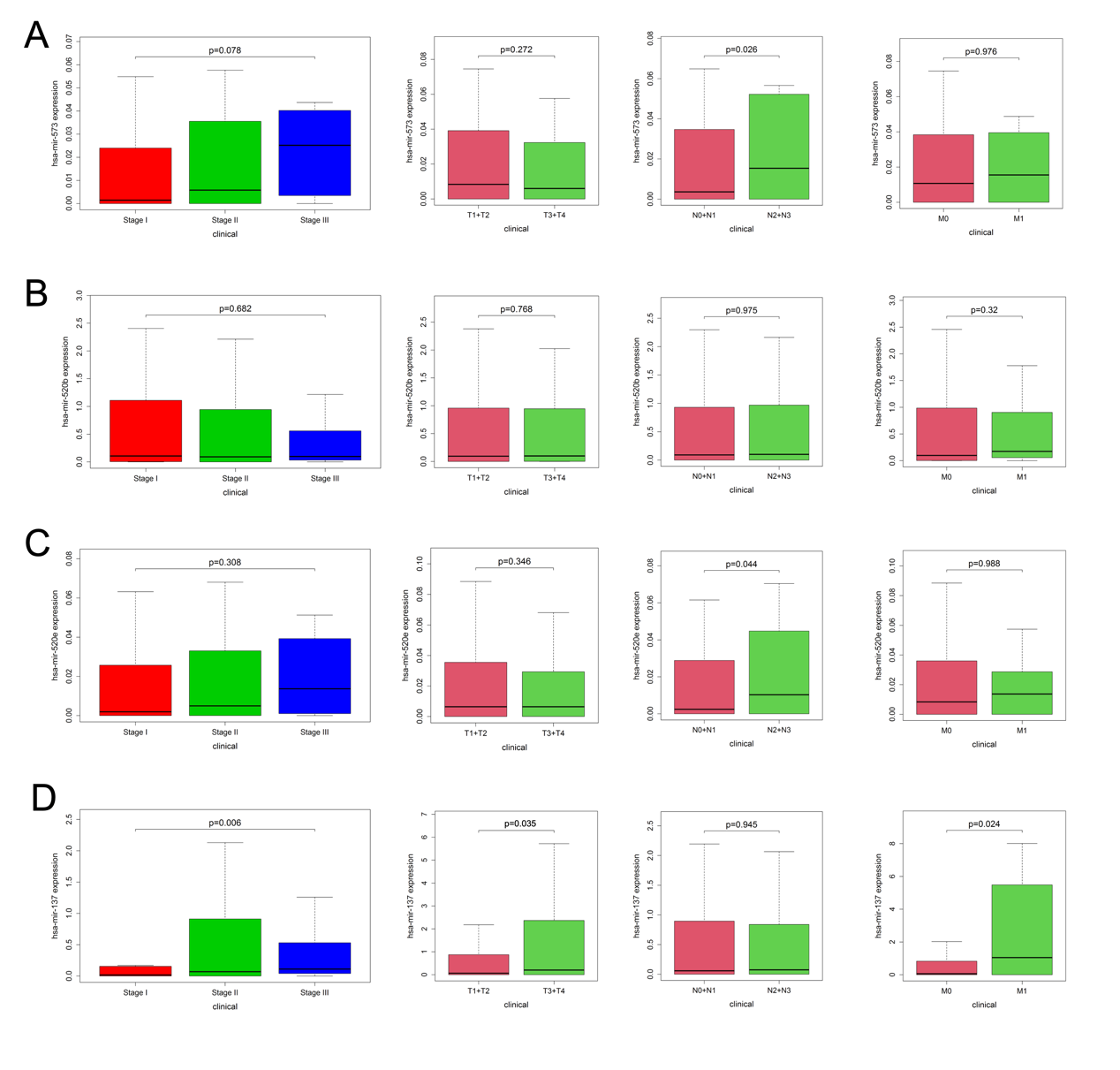
**

**Figure S4.** The Kaplan-Meier survival analysis about Luminal A-like patients with hsa-miR-944 high and low expression level.

**Figure S5.** The Kaplan-Meier survival analysis of 5 subtype BC patients with the different expression levels of hsa-miR-944 and HNRNPC.

**
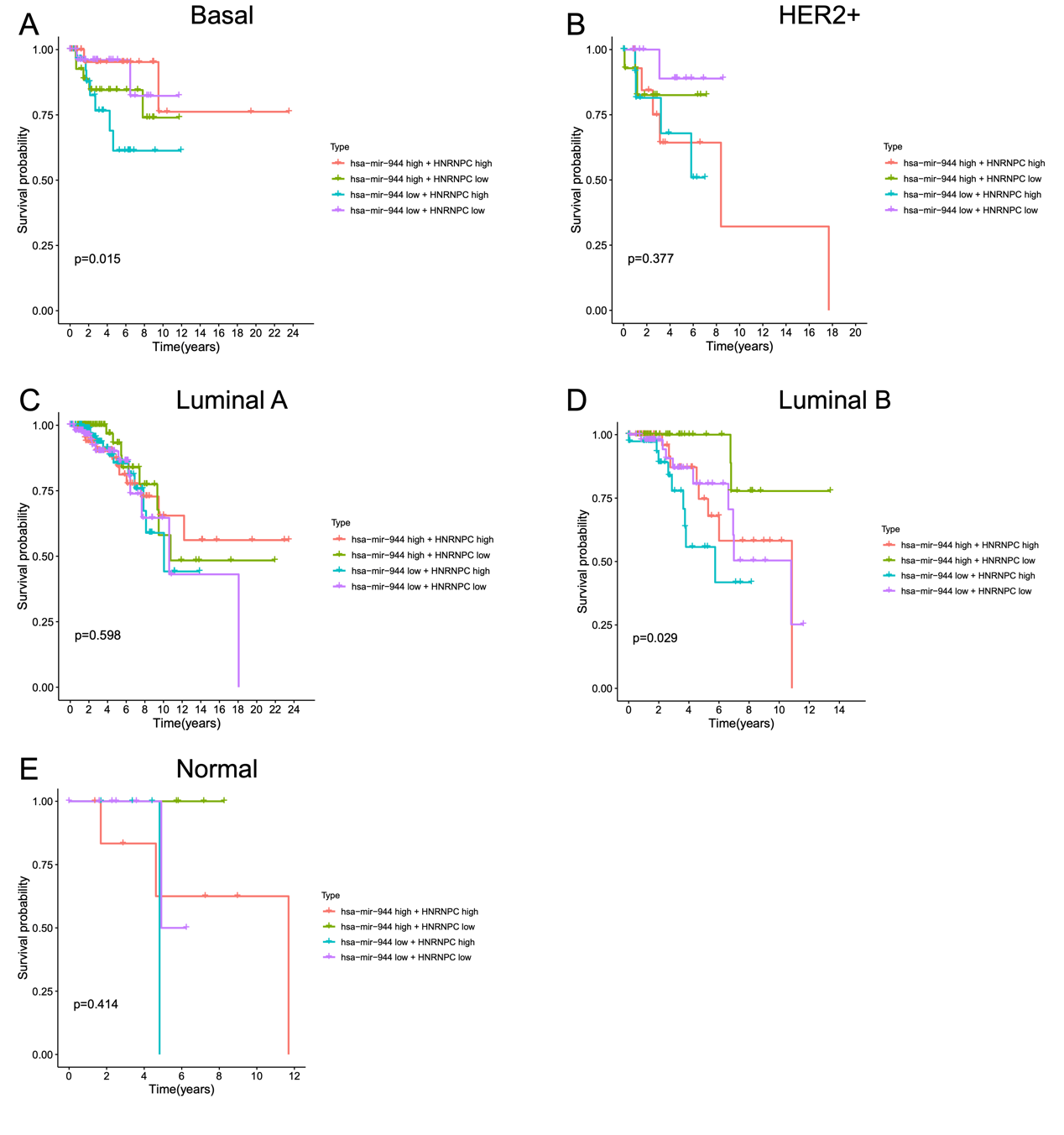
**

**Figure S6.** The CCK8 assay indicated the proliferation of MCF-7 cells (**A**) and MDA-MB-231 cells (**B**) after transfection with HNRNPC siRNAs or negative control.

**
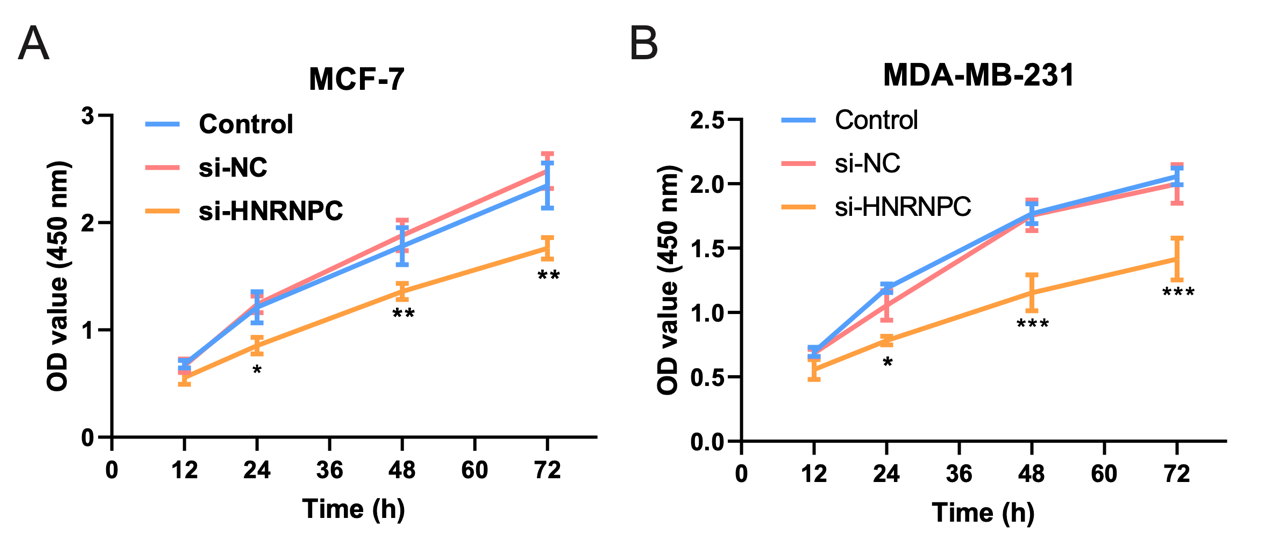
**

## 2. Supplementary Tables

**Table S1.** siRNA sequences for HNRNPC.

| Gene | sense 5’-3’ | antisense 5’-3’ |
| --- | --- | --- |
| Si-NC | CAGAAGAATGGTACAAATCCAAG | CTTCGTTCAGTATGTTAATCGT |
| si-HNRNPC-1 | CAACGGGACUAUUAUGAUA | UAUCAUAAUAGUCCCGUUG |
| si-HNRNPC-2 | GCGCUUGUCUAAGAUCAAAUU | AAUUUGAUCUUAGACAAGCGC |
| si-HNRNPC-3 | GCGCUUGUCUAAGAUCAAAUU | AAUUUGAUCUUAGACAAGCGC |

**Table S2.** Primers for qRT-PCR.

| Gene | sense 5’-3’ | antisense 5’-3’ |
| --- | --- | --- |
| has-circRNA-0001625 | CTCGAGATTTTTGTATTATTAACTGTAGT | GCGGCCGCTTAGCAGGAAGGCACTATT |
| HNRNPC | GCCAGCAACGTTAC CAACAA | TGAACAGAGCA GCCCACAAT |
| miR-944 | GCACTCCTAAAATTATTGTACATCG | TATGGTTGTTCACGACTCCTTCAC |
| U6 | CTCGCTTCGGCAGCACATATACT | ACGCTTCACGAATTTGCGTGTC |

**Table S3.** Summary of clinical characteristics of TCGA-BC dataset.

| **Characteristic** | **TCGA-BC data set (n = 1053)** |
| --- | --- |
| **Vital status, n (%)** |  |
| Alive | 911 (86.5) |
| Dead | 142 (13.5) |
| **Age, n (%)** |  |
| < 65 | 730 (69.3) |
| ≥ 65 | 323 (30.7) |
| **WHO-Stage, n (%)** |  |
| Ⅰ | 172 (16.3) |
| Ⅱ | 597 (56.7) |
| Ⅲ | 240 (22.8) |
| Ⅳ | 20 (1.9) |
| X | 13 (1.2) |
| Unknow | 11 (1.1) |
| **AJCC-T stage, n (%)** |  |
| T1 | 270 (25.6) |
| T2 | 614 (58.3) |
| T3 | 129 (12.3) |
| T4 | 37 (3.5) |
| TX | 3 (0.3) |
| **AJCC-N stage, n (%)** |  |
| N0 | 484 (46.0) |
| N1 | 354 (33.6) |
| N2 | 119 (11.3) |
| N3 | 76 (7.2) |
| NX | 20 (1.9) |
| **AJCC-M stage, n (%)** |  |
| M0 | 876 (83.2) |
| M1 | 22 (2.1) |
| MX | 155 (14.7) |

**Table S4.** The expression of 5 differentially expressed miRNAs in different GEO datasets.

| miRNA | GEO series | log_2_FC | P Value | Status |
| --- | --- | --- | --- | --- |
| hsa-miR-137 | GSE31309 | -0.0347365 | 0.081 | Down |
| hsa-miR-137 | GSE44124 | 0.0857038 | 0.085 | Up |
| hsa-miR-137 | GSE45498 | 0.045403 | 0.002 | Up |
| hsa-miR-520b | GSE31309 | 0.0668697 | 0.066 | Up |
| hsa-miR-520b | GSE44124 | 0.1319451 | 0.032 | Up |
| hsa-miR-520b | GSE45498 | -0.189728 | 0.011 | Down |
| **hsa-miR-944** | **GSE31309** | **-0.1099319** | **0.046** | **Down** |
| **hsa-miR-944** | **GSE44124** | **-0.0455618** | **0.027** | **Down** |
| **hsa-miR-944** | **GSE45498** | **-0.115973** | **0.033** | **Down** |
| hsa-miR-520e | GSE31309 | 0.1512685 | 0.034 | Up |
| hsa-miR-520e | GSE44124 | 0.0312644 | 0.043 | Up |
| hsa-miR-520e | GSE45498 | -0.530457 | 1.80E-04 | Down |
| hsa-miR-573 | GSE31309 | 0.1663451 | 0.023 | Up |
| hsa-miR-573 | GSE44124 | 0.0665785 | 0.045 | Up |
| hsa-miR-573 | GSE45498 | 0.472111 | 2.50E-04 | Up |

**Table S5.** The expression of 2 differentially expressed mRNAs in different GEO datasets.

| miRNA | GEO series | log_2_FC | P value | Status |
| --- | --- | --- | --- | --- |
| HNRNPC | GSE21422 | 0.248 | 0.046 | Up |
| YTHDF3 | GSE21422 | 0.846 | 0.019 | Up |
| HNRNPC | GSE59246 | 0.442 | 0.004 | Up |
| YTHDF3 | GSE59246 | 0.304 | 0.006 | Up |
| HNRNPC | GSE10797 | 0.288 | 0.003 | Up |
| YTHDF3 | GSE10797 | -0.241 | 0.545 | Down |
| HNRNPC | GSE38959 | 0.739 | 0.002 | Up |
| YTHDF3 | GSE38959 | 0.397 | 0.022 | Up |
